# A cohort study of serum 25-hydroxyvitamin D levels and the risk of hyperlipidaemia in adults

**DOI:** 10.3389/fnut.2024.1492621

**Published:** 2025-01-24

**Authors:** Zi-yue Liu, Sha Liu, Xue Yao, Chun-yang Wang, Yunhao Song, Yan-ming Bi, Jin-Xiu Wang, Yang Li, Ta-la Shi, Wei Mi, Caiyun Chen, Zhi-Yong Hu

**Affiliations:** ^1^School of Public Health, Binzhou Medical University, Yantai, China; ^2^Yantai Affiliated Hospital of Binzhou Medical College, Yantai, China; ^3^Occupation of Chinese Center for Disease Control and Prevention, Beijing, China

**Keywords:** vitamin D, hyperlipidaemia, cohort study, 25(OH)D, adults

## Abstract

**Objective:**

This study aims to investigate the potential association between serum 25(OH)D levels and the risk of hyperlipidemia in adults through a prospective cohort study conducted in Zhejiang Province.

**Methods:**

Baseline surveys and follow-up studies were conducted to collect and analyze follow-up data over a three-year period. Vitamin D deficiency was defined as 25(OH)D < 20 ng/mL, insufficiency as 20–29 ng/mL, and sufficiency as 25(OH)D ≥ 30 ng/mL. Hyperlipidemia or dyslipidemia was defined as the presence of hypercholesterolemia, hypertriglyceridemia, or both. The relationship between demographic characteristics and the incidence of hyperlipidemia among the study participants was explored.

**Results:**

A total of 1,210 participants were included in this study, with 43.80% being male. The mean age of the participants was 51.84 ± 14.37 years, and the average serum 25(OH)D level was 25.89 (21.50, 29.82) ng/mL. A significant difference in the proportion of vitamin D deficiency was observed between males and females (22.06% vs. 10.94%, *p* < 0.001). Vitamin D deficiency and insufficiency were prevalent among the middle-aged and elderly population (78.24%). Significant differences were found between the two groups in multiple sociodemographic variables, behavioral factors, and metabolic risk factors (*p* < 0.05). The incidence of hyperlipidemia among vitamin D-deficient individuals was 1.612 times higher than that among vitamin D-sufficient individuals (95% confidence interval [CI]: 1.228–2.116; *p* < 0.001). After fully adjusting for confounding factors, the multivariate-adjusted hazard ratio (HR) was 1.572 (95% CI: 1.187–2.08; *p* = 0.002), indicating a difference in the incidence of hyperlipidemia across different serum vitamin D levels.

**Conclusion:**

This cohort study reveals a significant association between serum 25(OH)D levels and the incidence of hyperlipidemia. Additionally, lifestyle factors associated with vitamin D deficiency are also correlated with the incidence of hyperlipidemia. These findings provide further evidence for improving blood lipid profiles through adjustments in vitamin D intake or related lifestyle modifications.

## 1 Introduction

Hyperlipidemia, as a chronic metabolic disorder characterized by abnormal lipid metabolism, exhibits a complex pathogenesis involving interactions between genetic and environmental factors (1, 2). Adverse dietary patterns, particularly excessive intake of high-fat and high-calorie foods and nutritional imbalances, coupled with lifestyle changes such as lack of regular exercise and irregular sleep patterns, along with specific medical interventions like drug side effects, collectively contribute to the development of hyperlipidemia (3, 4). This condition has emerged as a global public health challenge, not only severely impacting individuals’ quality of life but also significantly increasing the risk of severe complications such as cardiovascular disease, diabetes, and metabolic syndrome, thereby imposing a substantial burden on national healthcare systems (5). In recent years, with shifts in lifestyle, the incidence of hyperlipidemia has risen markedly (6). Data indicates that the prevalence of hypercholesterolemia among adults in the Americas and Europe is as high as 47.7 and 53.7%, respectively (7), while the prevalence of dyslipidemia among adults aged 65 and older in the United States has reached 60.3% (8). A recent meta-analysis from Malaysia also revealed high prevalence rates for various subtypes of dyslipidemia, emphasizing the global ubiquity of hyperlipidemia and the urgency for in-depth research (9).

Vitamin D (calciferol), a fat-soluble steroid hormone, primarily exists in the forms of ergocalciferol (Vitamin D_2_), derived from plant-based foods, and cholecalciferol (Vitamin D_3_), sourced from animal-based foods and endogenously synthesized in the epidermis under UVB light (10). In circulation, vitamin D binds primarily to vitamin D-binding protein (VDBP) and is transported to the liver for the initial hydroxylation to produce the main circulating metabolite, 25(OH)D (11). 7-dehydrocholesterol is converted into the vitamin D3 precursor by microsomal cytochrome P450 (12). Subsequently, 25(OH)D is transported to the kidneys or other extrarenal tissues (VAN ETTEN and MATHIEU (13)) for further hydroxylation into the biologically active form, 1,25-dihydroxyvitamin D [1,25(OH)2D] (14). Vitamin D is not only crucial for bone health (15), but also participates in regulating immune responses, cell proliferation and differentiation, and cardiovascular physiology among various biological processes (14). Recently, the association between vitamin D deficiency and an increased risk of metabolic, immune, and various chronic diseases has garnered considerable attention (16, 17).

When exploring the complex relationship between vitamin D levels and lipid metabolism, existing studies provide mixed evidence. Some studies have found that lower concentrations of 25(OH)D correlate with higher total and abdominal fat levels (18), and vitamin D deficiency is associated with elevated triglyceride (TG) concentrations in obese adults (19). A potential correlation between vitamin D deficiency and obesity may arise because adipose tissue can sequester vitamin D or because obese individuals may be less inclined to expose themselves to sunlight, thereby reducing skin synthesis of vitamin D. Numerous studies have pointed out the association between vitamin D deficiency and overweight, which further exacerbates the risk of lipid metabolism disorders (20, 21). Additionally, a potential correlation between vitamin D deficiency and increased total cholesterol (TC) levels and decreased low-density lipoprotein cholesterol (LDL-C) levels has been observed (22, 23). However, other studies have reported different trends, such as Ponda et al. (24) finding that patients with optimal 25(OH)D concentrations had lower LDL-C and TG concentrations but higher high-density lipoprotein cholesterol (HDL-C) concentrations. These discrepancies may stem from differences in study design, sample selection, and adjustment for confounding factors (25, 26).

This study aims to conduct a well-designed cohort study to delve into the potential association between serum 25(OH)D levels and the risk of hyperlipidemia in adults. By systematically analyzing the impact of different vitamin D concentrations on lipid metabolism patterns in hyperlipidemia patients, we hope to provide scientific support for the prevention and treatment of hyperlipidemia (27, 28). Although the specific mechanisms by which vitamin D regulates blood lipids are not fully understood, its positive effects are widely recognized (29, 30). Given the wide availability, cost-effectiveness, and safety of vitamin D, its potential as a blood lipid-regulating supplement is noteworthy (31). However, more in-depth research is needed regarding optimal dosage and intervention timing. This study employs a prospective cohort design to assess the relationship between serum vitamin D concentrations and the incidence of hyperlipidemia in the general population, aiming to validate and deepen the knowledge in this field, open new perspectives for the prevention and treatment of hyperlipidemia, and provide a scientific basis for future clinical practice.

## 2 Materials and methods

### 2.1 Study population

A multi-stage sampling method was employed for sample recruitment. Two communities/administrative villages were selected from 16 streets/towns in the Lishui area of southeastern Zhejiang Province using random integer sampling. Then, the corresponding number of participants was randomly selected from each community. The specific sampling approach was as follows. In the first step, cluster sampling was employed, with all communities/administrative villages within each street/township being assigned numerical labels. Two communities/administrative villages were selected through a random drawing process. In the second step, systematic sampling was utilized to select survey participants from the chosen communities/administrative villages. This involved sorting households by house number and using a random number generator to select the first household. Subsequent sample households were determined at intervals based on the total number of households in the community and the desired sample size. All household members over 18 years of age in the sampled households were selected as survey subjects. The sample of the study included 3,828 adult permanent residents.

From this larger sample, subjects were selected to participate in the current study. Subjects for the current study were selected based on the following inclusion criteria: (1) no hyperlipidaemia: serum TC < 6.2 mmol/L (240 mg/dL), serum TG < 2.3 mmol/L (200 mg/dL), serum LDL-C < 4.1 mmol/L (160 mg/dL) and serum HDL-C ≥ 1.0 mmol/L (40 mg/dL) (32), (2) no malignant diseases, chronic liver disease or chronic kidney disease; (3) no hyperlipidaemia drug treatment; (4) no vitamin D supplementation. At the end of the follow-up period, a total of 1,210 subjects were included in the statistical analysis. This study was designed as a cohort study, including baseline measurements and follow-up measurements over a three-year period. The data included survey data, anthropometry data and laboratory testing. All subjects provided written informed consent and the study was performed in strict accordance with the Helsinki Declaration. This topic has been approved by the Ethics Committee of Soochow University (No. ECSU-20160001).

### 2.2 Instruments and equipment

After fasting for 12 h, 5 mL of peripheral venous blood was collected from each subject. The blood was centrifuged at 3,000 r/min for 10 min, and the upper serum was taken to detect related indicators. Fasting blood glucose levels were measured by the hexokinase method using a fully automatic biochemical analyzer (COBAS c702, Roche Diagnostics, Mannheim, Germany). The levels of TG, TC, HDL-C and LDL-C were measured by the enzymatic method using an automatic biochemical analyzer (COBAS e601, Roche Diagnostics Co., Ltd., Mannheim, Germany). Serum 25(OH)D concentrations were determined by chemiluminescence immunoassays using an automatic chemiluminescence immunoassay analyzer (ADVIA Centaur XP, Siemens Medical Diagnostics, New York, United States). The intra-and inter-assay coefficients of variation for this method were <5%. These analyses were completed by Hangzhou Dean Medical Laboratory Centre Co., Ltd.

### 2.3 Baseline data collection

Standard questionnaires were used to collect demographic data (age, gender, area of residence, etc.), socioeconomic status (education level), smoking habits (current smoking, smoking cessation, or never smoking), drinking status (current drinking, smoking cessation, or never drinking), physical activity, family income, marital status, cognitive attitude toward chronic diseases, family history of hypertension and family history of diabetes. The education level is divided into four groups: no school, primary school, junior high school, college and above. This study used the World Health Organization’s physical activity recommendations (at least 150 min of moderate activity per week or the same amount of exercise) as a reference value for regular exercise. Family history of hypertension was defined as any first-degree relative (mother, father, sister, or brother) diagnosed with hypertension, and family history of diabetes was defined as any first-degree relative (mother, father, sister, or brother) diagnosed with diabetes.

### 2.4 Measurement of physical parameters

Physical examinations were performed in the early morning on an empty stomach and included measurements of height, weight, waist circumference and blood pressure. Height was measured by a height meter with a length of 2.0 m and an accuracy of 0.1 cm. Body weight is measured by a weight meter with a maximum weight of 150 kg and an accuracy of 0.1 kg. Blood pressure was measured using a standard mercury sphygmomanometer. Blood pressure was measured in millimeters of mercury, with an accuracy of up to 1 mm. After the subjects rested for 15 min, their blood pressure was measured three times in a relaxed seated position. There was a 5-min break between each measurement. The average of the three measurements was used for analysis. Hypertension was defined as systolic blood pressure (SBP) ≥ 140 mmHg and/or diastolic blood pressure (DBP) ≥ 90 mmHg and/or taking antihypertensive drugs and/or diagnosed with hypertension by a medical unit at or above the county level (33). Body mass index (BMI) was calculated as weight divided by the square of height (BMI = kg/m^2^). BMI ≥ 28 kg/m^2^ was defined as obese, 24 kg/m^2^ ≤ BMI < 28 kg/m^2^ as overweight and 18.5 kg/m^2^ ≤ BMI < 24 kg/m^2^ as normal (34).

### 2.5 Follow-ups and study outcome

All subjects were followed up by strictly trained community physicians. Data on relevant hyperlipidaemia events during the follow-up period were collected. The occurrence of hyperlipidaemia was the primary endpoint of this study. New-onset hyperlipidaemia was defined as a self-reported history of physician diagnosis during follow-up and/or receiving medication for hyperlipidaemia and/or serum TC ≥ 6.2 mmol/L (240 mg/dL) and/or serum TG ≥ 2.3 mmol/L (200 mg/dL) and/or serum LDL-C ≥ 4.1 mmol/L (160 mg/dL) and/or serum HDL-C < 1.0 mmol/L (40 mg/dL) (35).

### 2.6 Index division standards

(1) According to the Chinese Guidelines for the Prevention of Dyslipidaemia in Adults (2016 Edition) (32), serum TC ≥ 6.2 mmol/L (240 mg/dL) was defined as hypercholesterolemia, serum TG ≥ 2.3 mmol/L (200 mg/dL) was defined as hypertriglyceridemia, serum LDL-C ≥ 4.1 mmol/L (160 mg/dL) was defined as LDL-hypercholesterolemia, and serum HDL-C < 1.0 mmol/L (40 mg/dL) was defined as HDL-hypocholesterolemia. Hypercholesterolemia, hypertriglyceridemia, LDL-hypercholesterolemia or HDL-hypocholesterolemia were defined as hyperlipidaemia or dyslipidaemia.

(2) According to the diagnostic criteria of hypertension of the National Health Commission: (1) In the absence of antihypertensive drugs, the clinic blood pressure was measured three times on different days, SBP ≥ 140 mmHg and/or DBP ≥ 90 mmHg. Simple systolic hypertension was defined as SBP ≥ 140 mmHg and DBP < 90 mmHg; (2) The patient has a history of hypertension and is currently using antihypertensive drugs. Although the blood pressure is lower than 140/90 mmHg, it should still be diagnosed as hypertension (33).

(3) According to the Endocrine Society’s Clinical Practice Guidelines, serum 25(OH)D deficiency was defined as 25(OH)D < 20 ng/mL, insufficiency as (20–29 ng/mL), and sufficiency was defined as 25(OH)D ≥ 30 ng/mL.

### 2.7 Quality control

To ensure standardization of the project and obtain high-quality survey data, before the implementation of the survey, the community doctors, public health doctors, nursing staff and questionnaire survey personnel were uniformly trained. After the implementation of the project, on-site quality control was carried out, and the quality of the survey data, physical measurements, blood sample collection and laboratory testing were dynamically monitored in real time. When a quality problem was found, timely feedback and correction were performed to prevent the diffusion of the problem. All data are input by a unified program, and double data entry is used to check data logic and abnormal data.

### 2.8 Statistical analysis

EpiData 3.1 software was used for double data entry, logical error correction and consistency testing. Normally distributed data were expressed as the mean ± standard deviation ( $\bar{x}$ ± s), non-normally distributed data were expressed as the median and interquartile range [M(QR)] and categorical variables were expressed as frequencies (%). Analysis of variance (ANOVA) and the Chi-square test were used to analyze the differences in quantitative variables between 25(OH)D level groups. Between-group differences in qualitative variables were tested by the Chi-square test. A Cox proportional hazard model was used to evaluate HR and 95% CI for the relationship between baseline serum 25(OH)D levels and hyperlipidaemia. In model 1, only serum 25(OH)D was included as an independent predictor. Age, gender and BMI levels were also included in model 2. Model 3 was based on model 2 with further adjustment for the following variables: residence area (rural or urban), education level, SBP, DBP, smoking, alcohol consumption, physical activity and family history of hypertension. The median serum 25(OH)D value for each group was input into the model as a continuous variable for trend testing. Then, the association between serum 25(OH)D levels and the risk of hyperlipidaemia was examined in subgroups stratified by age, sex, BMI, area of residence, education, SBP, DBP, smoking, alcohol consumption, physical activity and family history of hypertension. The subjects were divided into two groups according to a 25(OH)D cut-point of 30 ng/mL. All data were analyzed using SPSS version 26.0, and statistical significance was defined as a two-tailed *p* < 0.05.

## 3 Results

### 3.1 Demographic characteristics and incidence of hyperlipidaemia

The study sample comprised 1,210 individuals, among which, 43.80% were male (530 subjects) and 56.19% were female (680 subjects). The average age of the subjects was 51.84 ± 14.37 years. As shown in Table 1, there were 368 new cases of hyperlipidaemia (167 males and 201 females) during the three-year follow-up period, and the cumulative incidence of hyperlipidaemia was 30.41%. Specifically, the cumulative incidence of hyperlipidaemia was 31.51% in males and 29.56% in females. There was no statistically significant difference in the prevalence of hyperlipidaemia between male and female subjects (*p* > 0.05). In the relationship between age and the incidence of hyperlipidemia shown in Table 1, with the increase of age, the incidence of hyperlipidemia in male and female participants showed a significant upward trend, and when the age reached a certain stage, this upward trend did not continue, but there was a slight decline in the reversal. The incidence of hyperlipidaemia increased the fastest among men in the 30–39 years age group.

**Table 1:** Incidence of hyperlipidemia in different sex and age groups.

Age group (years)	Baseline number	Hyperlipidemia	Follow-up years	Cumulative incidence (%)	Incidence density (per 1,000 person-years)
Whole crowd					
18-	80	14	26.88	17.50	520.91
30-	180	51	80.86	28.33	630.73
40-	277	84	134.64	30.32	623.89
50-	314	111	201.07	35.35	552.04
60-	236	72	122.53	30.51	587.61
70-	95	32	52.82	33.68	605.78
80-	28	4	8.65	14.29	462.36
Total	1,210	368	3153.45	30.41	116.70
Male					
18-	27	5	75.63	18.52	66.11
30-	67	34	148.11	50.75	229.55
40-	110	42	280.25	38.18	149.87
50-	136	39	363.45	28.68	107.31
60-	118	29	321.64	24.58	90.16
70-	56	18	149.16	32.14	120.67
80-	16	0	48.00	0.00	0.00
Total	530	167	1386.24	31.51	120.47
Female					
18-	53	9	149.25	16.98	60.30
30-	113	17	327.04	15.04	51.98
40-	167	42	455.50	25.15	92.21
50-	178	72	456.85	40.45	157.60
60-	118	43	304.66	36.44	141.14
70-	39	14	98.37	35.90	142.32
80-	12	4	32.65	33.33	122.51
Total	680	201	1824.32	29.56	110.18

We recorded the baseline survey time and follow-up time of each participant. Participants were tracked at different times during the three-year period. After completing the calculation of the number of follow-up years at the individual level, summary statistics were performed to calculate the total follow-up years and average follow-up years of the entire cohort.

### 3.2 Comparison of the baseline characteristics of subjects classified by vitamin D status

Among the 1,210 participants, the overall mean serum 25(OH)D level was 25.87 ± 6.56 ng/mL, and the respective ratios of vitamin D deficiency (<20 ng/mL) and insufficiency (20–29 ng/mL) were 17.19 and 58.76%. According to Table 2, the rates of vitamin D deficiency and insufficiency in male subjects were 10.94% (58 people) and 52.45% (278 people), respectively, and the rates of vitamin D deficiency and insufficiency in female subjects were 22.06% (150 people) and 63.68% (433 people), respectively. Vitamin D deficiency was more common in women than in men (22.06% vs. 10.96%). With increases in age, the vitamin D deficiency rate showed an upward trend; only 21.43% of the elderly subjects aged over 80 years had sufficient vitamin D. The rates of vitamin D deficiency and insufficiency among rural residents were 12.93% (67 people) and 57.92% (300 people), respectively. The rates of vitamin D deficiency and insufficiency among urban residents were 20.36% (141 people) and 59.39% (411 people), respectively. The rates of vitamin D deficiency and insufficiency in subjects with BMI < 24 kg/m2 were 16.80% (128 people) and 26.90% (205 people), respectively. The rate of vitamin D deficiency was higher in women and participants living in urban areas (*p* < 0.01). Aside from baseline age, education level, SBP, DBP, dyslipidaemia and family history of hypertension, the differences between other sociodemographic, behavioral and metabolic risk factors and vitamin D levels were statistically significant (all *p* < 0.05, Table 3).

**Table 2:** Incidence of hyperlipidemia in different sex and age groups.

Characteristic	Vitamin D deficiency	Vitamin D insufficiency	Vitamin D sufficiency	Total
Male, *n*(%)				
18-	2(7.41)	16(59.26)	9(33.33)	27
30-	8(11.94)	32(47.76)	27(40.3)	67
40-	12(10.91)	60(54.55)	38(34.55)	110
50-	18(13.24)	64(47.06)	54(39.71)	136
60-	9(7.63)	69(58.47)	40(33.9)	118
70-	6(10.71)	30(53.57)	20(35.71)	56
80-	3(18.75)	7(43.75)	6(37.5)	16
Total	58(10.94)	278(52.45)	194(36.6)	530
Female, *n*(%)				
18-	10(18.87)	36(67.92)	7(13.21)	27
30-	29(25.66)	67(59.29)	17(15.04)	67
40-	31(18.56)	112(67.07)	24(14.37)	110
50-	31(17.42)	120(67.42)	27(15.17)	136
60-	25(21.19)	75(63.56)	18(15.2)	118
70-	16(41.03)	19(48.72)	4(10.26)	56
80-	8(66.67)	4(33.33)	0(0)	16
Total	150(22.06)	433(63.68)	97(14.26)	530

**Table 3:** Baseline characteristics of the study population (*n* = 1,210).

Characteristic	Total	Serum 25(OH)D concentrations (ng/ml)			*F*/*χ*^2^	*P*
	(*n* = 1,210)	<20(*n* = 208)	20–29(*n* = 711)	≥30(*n* = 291)		
25(OH)D	25.87 ± 6.56	16.16 ± 2.96	25.32 ± 2.78	34.15 ± 4.16		
Age (years)	51.84 ± 14.37	52.92 ± 15.74	51.39 ± 14.07	52.17 ± 14.04	1.01	0.365
Sex (men), *n*(%)	530(43.80)	58(10.94)*	278(52.45)	194(36.60)	89.598	<0.001
(Women), *n*(%)	680(56.20)	150(22.06)	433(63.68)	97(14.26)		
District (rural), *n*(%)	518(42.81)	67(12.93)*	300(57.92)	151(29.15)	19.453	<0.001
(Urban), *n*(%)	692(57.19)	141(20.38)	411(59.39)	140(20.23)		
Education, *n*(%)					7.148	0.307
No school	210(17.36)	36(17.14)	129(61.43)	45(21.43)		
Primary school	328(27.11)	56(17.07)	182(55.49)	90(27.44)		
Middle school	611(50.50)	105(17.18)	358(58.59)	148(24.22)		
Junior college or higher	61(5.04)	11(18.03)	42(68.85)	8(13.11)		
BMI (kg/m^2^)	23.28 ± 3.24	23.34 ± 3.34	23.44 ± 3.26*	22.86 ± 3.09	3.364	0.035
<24	762(62.98)	128(61.54)	429(60.34)	205(70.45)	9.414	0.052
24–27.9	346(28.60)	62(29.81)	219(30.80)	65(22.34)		
≥28	102(8.43)	18(8.65)	63(8.86)	21(7.22)		
TC	4.95 ± 0.72	5.13 ± 0.72	4.95 ± 0.72*	4.84 ± 0.69	9.978	<0.001
TG	1.25 ± 0.45	1.32 ± 0.46	1.26 ± 0.45*	1.17 ± 0.46	6.674	0.001
LDL-C	2.84 ± 0.60	2.94 ± 0.63	2.85 ± 0.60*	2.75 ± 0.59	5.726	0.003
HDL-C	1.58 ± 0.34	1.66 ± 0.48	1.57 ± 0.31*	1.57 ± 0.31	6.078	0.002
Systolic pressure	125.01 ± 18.77	126.70 ± 20.32	124.14 ± 18.45	125.93 ± 19.04	1.954	0.142
Diastolic pressure	78.72 ± 10.21	78.62 ± 11.13	78.90 ± 10.11	78.36 ± 9.81	0.298	0.743
Family history of hypertension, *n*(%)	258(21.32)	46(17.83)	46(17.83)	46(17.83)	0.989	0.610
Dyslipidemia, *n*(%)	53(4.38)	12(5.77)	30(4.22)	11(3.78)	7.150	0.128
Current smoker, *n*(%)	267(22.08)	39(18.84)	146(20.53)*	82(28.18)	8.539	0.014
Current drinker, *n*(%)	370(30.63)	41(19.81)	217(30.52)*	112(38.62)	20.131	<0.001
Sufficient physical activity, *n*(%)	292(24.17)	56(27.18)	185(26.02)*	51(17.53)	9.357	0.009

*There was a significant difference compared with the 25(OH) D sufficient group [serum 25(OH)D ≥ 30 ng/mL]; TG, triglyceride; TC, total cholesterol; HDL-C, high density lipoprotein cholesterol; LDL-C, low density lipoprotein cholesterol.

### 3.3 The relationship between serum 25(OH)D levels and hyperlipidaemia

As shown in Table 4, when the clinical classification of vitamin D status was used, the subjects with sufficient vitamin D (≥30 ng/mL) were used as a reference. The incidence of hyperlipidemia in vitamin D deficiency (20–29 ng/mL) was statistically different (*p* < 0.05), and the incidence of hyperlipidemia was significantly higher than that of vitamin D sufficient subjects. When using the clinical classification of vitamin D status, compared with the survey of vitamin D sufficiency (≥30 ng/mL), the incidence of hyperlipidemia in patients with vitamin D insufficiency (<30 ng/mL) was significantly increased (*p* < 0.05). According to the quartile of baseline 25(OH)D level, the study population was divided into four groups (Q1, Q2, Q3, and Q4). Taking the respondents in the Q4 group as a reference, the incidence of hyperlipidemia in the Q2 and Q3 groups was higher than that in the Q4 group (*p* < 0.05).

**Table 4:** Relationship between baseline serum 25(OH)D levels and the incidence of hyperlipidemia.

25(OH)D (ng/ml)	Events (%)	Model 1		Model 2		Model 3	
		HR(95% CI)	*P*	HR(95% CI)	*P*	HR(95% CI)	*P*
Clinical triad							
<20	208(60)	1.306(0.921–1.853)	0.134	1.309(0.915–1.873)	0.141	1.315(0.914–1.89)	0.140
20–29	711(242)	1.612(1.228–2.116)	0.001	1.581(1.197–2.087)	0.001	1.572(1.187–2.08)	0.002
≥30	291(66)	1.00	–	1.00	–	1.00	–
*P* for trend^†^		0.984(0.965–1.003)	0.100	0.985(0.965–1.004)	0.123	0.984(0.965–1.004)	0.123
Dichotomy							
<30	919(302)	1.540(1.180–2.010)	0.001	1.523(1.159–2.001)	0.003	1.519(1.130–2.001)	0.003
≥30	291(66)	1.00	–	1.00	–	1.00	–
P for trend^†^		0.953(0.925–0.982)	0.001	0.954(0.925–0.984)	0.003	0.954(0.925–0.984)	0.003
Quartile							
Q1(<21.50)	301(90)	1.280(0.940–1.743)	0.117	1.259(0.916–1.731)	0.156	1.265(0.916–1.746)	0.153
Q2(21.50–25.88)	303(109)	1.580(1.175–2.125)	0.002	1.565(1.156–2.119)	0.004	1.535(1.130–2.084)	0.006
Q3(25.89–29.82)	303(96)	1.392(1.027–1.888)	0.033	1.351(0.993–1.837)	0.055	1.350(0.989–1.843)	0.059
Q4(≥29.83)	303(73)	1.00	–	1.00	–	1.00	–
*P* for trend^†^		0.984(0.965–1.003)	0.107	0.985(0.966–1.005)	0.150	0.985(0.965–1.006)	0.153
Continuous^‡^	1,210(368)	0.987(0.972–1.003)	0.104	0.988(0.972–1.004)	0.139	0.987(0.971–1.004)	0.136

Model 1: no adjustment; model 2: adjust gender, age and BMI; model 3: adjust residence, education level, systolic blood pressure, diastolic blood pressure, smoking status, drinking status, physical activity and family history of hypertension.

HR, hazard ratio; CI, confidence interval.

^†^Test trends based on median variables in each group (medians for Q1, Q2, Q3, and Q4 were 18.55, 24.17, 27.92, and 32.88 ng/mL, respectively).

^‡^HR is the standard deviation (6.43 ng/mL) proportionally scaled to serum 25(OH)D levels.

After adjusting the potential confounding factors of age, gender and BMI, when the vitamin D status was classified into three clinical categories, the incidence of hyperlipidemia in vitamin D deficiency subjects was still significantly higher than that in vitamin D sufficient subjects (*p* < 0.05). When the clinical classification of vitamin D status was used, the investigation of vitamin D adequacy was used as a reference. There was a statistically significant difference in the incidence of hyperlipidemia between vitamin D insufficiency and sufficiency (*p* < 0.05), and the incidence of hyperlipidemia in vitamin D insufficiency was higher. According to the baseline 25(OH)D level quartiles, the study population was divided into four groups. The subjects in the Q4 group were used as a reference. There was a statistically significant difference in the incidence of hyperlipidemia between the subjects in the Q2 group and the Q4 group (*p* < 0.05).

After adjusting the potential confounding factors such as residence, education level, SBP, DBP, smoking status, drinking status, physical activity and family history of hypertension, when the vitamin D status was classified into three clinical categories, the subjects with sufficient vitamin D were used as a reference, and the incidence of hyperlipidemia in vitamin D deficiency was still high (*p* < 0.05). When using the clinical classification of vitamin D status, the incidence of hyperlipidemia in vitamin D deficiency and sufficient subjects was higher than that in vitamin D sufficient subjects (*p* < 0.05). According to the quartile of baseline 25(OH)D level, the study population was divided into four groups. Taking the respondents in the Q4 group as a reference, the incidence of hyperlipidemia in the Q4 group was lower than that in the Q2 group (*p* < 0.05).

As shown in Table 5, when analyzing the relationship between baseline serum 25-hydroxyvitamin D (25(OH)D) levels and the incidence of hyperlipidemia among individuals with normal weight, after excluding those who were overweight or obese, it was found that there was no statistically significant association between vitamin D and the risk of hyperlipidemia, regardless of the vitamin D clinical classification used. However, in the subset of overweight and obese individuals (Table 6), under the clinical binary classification of vitamin D status, those with vitamin D deficiency (<30 ng/mL) exhibited a significantly higher incidence of hyperlipidemia compared to those with sufficient vitamin D (≥30 ng/mL) (*p* < 0.05). Similarly, under the clinical ternary classification of vitamin D status, with those having sufficient vitamin D (≥30 ng/mL) as the reference group, individuals with vitamin D insufficiency (20–29 ng/mL) demonstrated a statistically significant difference in hyperlipidemia incidence (*p* < 0.05). When the study population was stratified into four groups based on quartiles of baseline 25(OH)D levels (Q1, Q2, Q3, and Q4), with Q4 serving as the reference group, the incidence of hyperlipidemia was higher in Q1, Q2, and Q3 compared to Q4 (*p* < 0.05).

**Table 5:** Relationship between baseline serum 25(OH)D levels and the incidence of hyperlipidemia (excluding overweight and obese people from normal-weight individuals).

25(OH)D	Events (%)	Model 1		Model 2		Model 3	
(ng/ml)		HR(95% CI)	*P*	HR(95% CI)	*P*	HR(95% CI)	*P*
Clinical triad							
<20	81(32)	0.982(0.612–1.576)	0.939	0.956(0.497–1.838)	0.892	0.999(0.549–1.817)	0.997
20–29	287(127)	1.125(0.780–1.623)	0.528	0.930(0.552–1.565)	0.784	1.024(0.625–1.677)	0.925
≥30	87(37)	1.00	–	1.00	–	1.00	–
*P* for trend^†^		0.989(0.965–1.015)	0.410	0.990(0.965–1.016)	0.451	0.990(0.965–1.017)	0.472
Dichotomy							
<30	368(159)	1.093(0.764–1.563)	0.626	0.935(0.561–1.558)	0.796	1.017(0.633–1.635)	0.944
≥30	87(37)	1.00	–	1.00	–	1.00	–
*P* for trend^†^		0.988(0.954–1.023)	0.508	0.988(0.953–1.024)	0.514	0.989(0.954–1.026)	0.550
Quartile							
Q1(<21.15)	114(45)	0.923(0.617–1.38)	0.697	0.898(0.513–1.570)	0.705	0.952(0.568–1.596)	0.851
Q2(21.16–25.38)	113(52)	1.086(0.737–1.602)	0.676	0.828(0.477–1.439)	0.504	0.955(0.565–1.612)	0.862
Q3(25.39–28.80)	113(49)	1.068(0.720–1.584)	0.742	1.074(0.618–1.865)	0.801	1.021(0.603–1.730)	0.937
Q4(≥28.81)	115(50)	1.00	–	1.00	–	1.00	–
*P* for trend^†^		0.993(0.969–1.018)	0.579	0.994(0.969–1.019)	0.625	0.994(0.969–1.020)	0.669
Continuous^‡^	455(196)	0.993(0.973–1.013)	0.490	0.993(0.973–1.014)	0.508	0.994(0.973–1.015)	0.585

Model 1: no adjustment; model 2: adjust gender, age and BMI; model 3: adjust residence, education level, systolic blood pressure, diastolic blood pressure, smoking status, drinking status, physical activity and family history of hypertension.

HR, hazard ratio; CI, confidence interval.

^†^Test trends based on median variables in each group (medians for Q1, Q2, Q3, and Q4 were 18.62, 23.86, 26.94, and 32.15 ng/mL, respectively).

^‡^HR is the standard deviation (6.00 ng/mL) proportionally scaled to serum 25(OH)D levels.

**Table 6:** Relationship between baseline serum 25(OH)D levels and the incidence of hyperlipidemia (overweight and obese people).

25(OH)D	Events (%)	Model 1		Model 2		Model 3	
(ng/ml)		HR(95% CI)	*P*	HR(95% CI)	*P*	HR(95% CI)	*P*
Clinical triad							
<20	127(28)	1.571(0.935–2.641)	0.088	1.637(0.957–2.802)	0.072	1.643(0.955–2.826)	0.073
20–29	424(115)	1.987(1.322–2.986)	0.001	2.052(1.351–3.118)	0.001	2.098(1.374–3.201)	0.001
≥30	204(29)	1.00	–	1.00	–	1.00	–
*P* for trend^†^		1.005(0.991–1.021)	0.475	1.005(0.989–1.020)	0.551	1.004(0.988–1.020)	0.660
Dichotomy							
<30	551(143)	1.889(1.267–2.816)	0.002	1.967(1.302–2.972)	0.001	2.013(1.323–3.061)	0.001
≥30	204(29)	1.00	–	1.00	–	1.00	–
*P* for trend^†^		1.012(0.992–1.032)	0.253	1.011(0.990–1.032)	0.309	1.010(0.989–1.032)	0.348
Quartile							
Q1(<21.73)	186(44)	1.821(1.115–2.975)	0.017	1.884(1.131–3.140)	0.015	1.894(1.128–3.181)	0.016
Q2(21.73–26.47)	191(51)	2.114(1.310–3.411)	0.002	2.201(1.351–3.585)	0.002	2.239(1.369–3.664)	0.001
Q3(26.48–30.45)	188(52)	2.287(1.419–3,684)	0.001	2.315(1.429–3.749)	0.001	2.360(1.448–3.847)	0.001
Q4(≥30.46)	190(25)	1.00	–	1.00	–	1.00	–
*P* for trend^†^		1.008(0.993–1.023)	0.313	1.007(0.992 ~ 1.023)	0.374	1.006-(0.9911.022)	0.441
Continuous^‡^	755(172)	1.005(0.993–1.017)	0.394	1.004(0.9921.017)	0.484	1.005(0.993–1.017)	0.394

Model 1: no adjustment; model 2: adjust gender, age and BMI; model 3: adjust residence, education level, systolic blood pressure, diastolic blood pressure, smoking status, drinking status, physical activity and family history of hypertension.

HR, hazard ratio; CI, confidence interval.

^†^Test trends based on median variables in each group (medians for Q1, Q2, Q3, and Q4 were 18.53, 24.42, 28.38, and 33.34 ng/mL, respectively).

^‡^HR is the standard deviation (6.86 ng/mL) proportionally scaled to serum 25 (OH)D levels.

After fully adjusting for potential confounding factors such as age, gender, and BMI, under the clinical ternary classification of vitamin D status, with those having sufficient vitamin D as the reference group, individuals with vitamin D insufficiency (20–29 ng/mL) still exhibited a significantly higher incidence of hyperlipidemia (*p* < 0.05). When using the clinical binary classification of vitamin D status, with those having sufficient vitamin D as the reference, there was a statistically significant difference in hyperlipidemia incidence between vitamin D-deficient and sufficient individuals (*p* < 0.05), with a higher incidence among the deficient group. Additionally, when stratifying the study population into four groups based on quartiles of baseline 25(OH)D levels and using Q4 as the reference, there was a statistically significant difference in hyperlipidemia incidence between Q2 and Q4 (*p* < 0.05). After fully adjusting for potential confounding factors such as residence, education level, systolic blood pressure, diastolic blood pressure, smoking status, alcohol consumption, physical activity, and family history of hypertension, the results remained consistent with the previous findings.

## 4 Discussion

A substantial body of literature has established a strong association between vitamin D and various disease states, including, but not limited to, type 2 diabetes, dyslipidemia, cardiovascular diseases, autoimmune disorders, certain cancers, metabolic syndrome, schizophrenia, and depression (CHEN (3, 36)). Clinical trial data, in particular, reveal a close relationship between 25(OH)D levels and lipid metabolism, indicating that elevated lipid levels can lead to a decrease in vitamin D levels, while vitamin D deficiency further exacerbates dyslipidemia and weight gain (37). Vitamin D deficiency has become a global public health issue, affecting individuals across all age groups, ethnicities, and socioeconomic statuses (38). Certain groups, such as individuals with obesity, the elderly, children, pregnant women, and postmenopausal women, exhibit especially high rates of vitamin D deficiency (39). Hyperlipidemia, as an increasingly prevalent metabolic disorder, not only poses a threat to individual health but also places a considerable burden on global healthcare systems (40, 41). For instance, by 2017, over 100 million people in the United States had been diagnosed with hypercholesterolemia, with 31 million adults experiencing elevated LDL-C levels (42). Therefore, investigating effective treatment strategies for hyperlipidemia, particularly regarding the potential role of vitamin D, is essential for managing lipid levels and improving patient outcomes.

Research has demonstrated that vitamin D plays a crucial role in reducing lipid levels and mitigating mortality risk (43), especially in patients with hypertension and type 2 diabetes (44). Theoretically, vitamin D not only exerts a direct influence on lipid levels but may also indirectly impact lipid metabolism by modulating serum parathyroid hormone and/or calcium balance (45). Specifically, sufficient vitamin D increases intracellular calcium levels in adipocytes, promoting the activity of fatty acid synthase, inhibiting lipolysis, and enhancing the storage of lipids within adipocytes (46). Additionally, vitamin D can inhibit macrophage migration and phagocytic activity, reducing the deposition of LDL-C (47). Conversely, as vitamin D levels decline, calcium absorption in the gastrointestinal tract diminishes (48), impairing the lipid storage function of adipocytes, which leads to the release of lipids into the bloodstream, causing elevated concentrations of TC, TG, and LDL-C, as well as a decrease in HDL-C levels (49). Studies from Australia (50) and animal models (51) further support that adequate vitamin D supplementation effectively inhibits hepatic steatosis and fibrosis, promotes hepatic calcium absorption, and thereby helps regulate lipid profiles.

However, it is worth noting that when we limited the analysis to individuals with normal weight, the results showed a very different pattern (73). We discovered that restricting the analysis to overweight and obese individuals can unveil a significant statistical correlation between vitamin D status and hyperlipidemia risk. In the clinical dichotomous classification of vitamin D status, individuals with vitamin D insufficiency (<30 ng/mL) exhibited a significantly higher incidence of hyperlipidemia compared to those with sufficient vitamin D (≥30 ng/mL) (*p* < 0.05). When further refining the classification into trichotomous categories, individuals with vitamin D levels in the insufficient range (20–29 ng/mL), using those with sufficient vitamin D as a reference, meanwhile demonstrated a statistical difference in hyperlipidemia incidence (*p* < 0.05). These observations strongly indicate that, in overweight and obese adults, the nutritional status of vitamin D may exert a notable impact on lipid metabolism, thereby increasing the risk of hyperlipidemia. To further explore this relationship, we grouped the study population based on quartiles of baseline 25(OH)D levels. The results showed that, using the highest quartile group (Q4) of 25(OH)D levels as a reference, individuals in the lower quartiles (Q1, Q2, and Q3) had significantly higher incidences of hyperlipidemia (*p* < 0.05). This trend analysis not only reinforces the negative correlation between vitamin D and hyperlipidemia risk but also suggests a potential dose–response effect of increasing vitamin D levels on reducing hyperlipidemia risk. Notably, this effect is particularly evident in overweight and obese populations, providing robust evidence to support vitamin D supplementation strategies tailored for these groups.

However, it is worth noting that when we expanded the analysis to individuals with normal body weight, the research findings presented a starkly different pattern. Regardless of the vitamin D clinical classification standard employed (i.e., dichotomous, trichotomous, or more detailed quartile classification), no statistically significant correlation was observed between baseline serum 25(OH)D levels and the risk of hyperlipidemia. This finding may suggest that, in adults with normal body weight, the nutritional status of vitamin D may not be a primary influencing factor in the development of hyperlipidemia, or its effect may be obscured by other more dominant metabolic factors. This result challenges the view that vitamin D is generally beneficial to lipid metabolism in some previous studies, and further emphasizes that there may be differences in the potential mechanism of vitamin D on lipid metabolism under overweight (74, 75).

The results of this study further confirm a positive association between vitamin D deficiency and the incidence of hyperlipidemia. When baseline 25(OH)D levels were considered as the sole predictor, vitamin D deficiency was significantly associated with an increased incidence of dyslipidemia. Even after extensive adjustment for confounding variables, a low vitamin D status remained significantly associated with dyslipidemia. Notably, the cumulative incidence of hyperlipidemia did not show a statistically significant difference between women and men, suggesting that the mechanisms underlying hyperlipidemia may transcend gender and are likely driven by similar fundamental physiological and genetic factors. However, in terms of vitamin D deficiency prevalence, a gender disparity was observed, with women exhibiting a higher frequency of vitamin D deficiency compared to men (22.06% vs. 10.94%), consistent with findings from a cross-sectional study in the Netherlands (52). This gender-specific variation may stem from differences in lifestyle, physiological characteristics, time spent outdoors, sun protection practices, and occupational activities between men and women (53). For instance, traditional or societal expectations may lead women to adopt sun protection measures or spend more time indoors, thereby reducing natural sunlight exposure—a primary pathway for endogenous vitamin D synthesis. Additionally, women experience unique life stages and hormonal changes, including menarche, potential pregnancy, breastfeeding, and contraceptive use, through to menopause, where the production of hormones such as estrogen and progesterone may predispose them to vitamin D deficiency (54). Furthermore, occupational differences could contribute to unequal sunlight exposure opportunities between genders. These factors collectively influence the efficiency of vitamin D synthesis in the skin, either directly or indirectly. Additionally, dietary habits—another crucial source of vitamin D—may also vary by gender. Women, in their pursuit of healthier eating, may inadvertently restrict foods rich in vitamin D, especially within specific dietary cultures or societal norms. In contrast, men may be less affected by these factors, or their dietary patterns may naturally include more vitamin D-rich foods. These hypotheses warrant further research, with detailed dietary surveys and lifestyle assessments needed to substantiate these findings.

With advancing age, the incidence of hyperlipidemia and vitamin D deficiency increases in both men and women, aligning with findings from previous literature (55). This age-related rise may be attributable to reduced outdoor activity, diminished gastrointestinal function, and imbalances in dietary intake and nutrition, which can lead to decreased vitamin D absorption and greater intracellular deposition of LDL-C in older adults (56). Interestingly, the incidence of hyperlipidemia tends to slightly decline among the elderly at certain advanced ages. This may be due to the premature attrition of high-risk individuals from the cohort because of the onset or progression of other health conditions, such as cardiovascular disease or diabetes, which reduces their representativeness in the study’s data on hyperlipidemia incidence (57). Such individuals may experience limited ability or willingness to participate due to health complications, thereby decreasing their representation in the cohort. Additionally, it is essential to consider the effects of aging on physiological function and metabolic processes (58, 59). With age, physiological functions, including lipid metabolism, undergo gradual decline, potentially impacting the incidence of hyperlipidemia either directly or indirectly (60). To further investigate this phenomenon, we plan to conduct a refined age-stratified analysis in future studies to elucidate similarities and differences in the mechanisms of hyperlipidemia onset across different age groups. Furthermore, we observed that individuals with lower educational attainment, lower BMI, or a family history of hyperlipidemia are more susceptible to both vitamin D deficiency and dyslipidemia. This trend may be associated with limited health literacy, imbalanced diets, inadequate nutrient intake, and genetic predispositions (61). First, individuals with lower educational levels may lack health knowledge and practices, such as regular monitoring of vitamin D levels and taking appropriate supplements, which could increase their risk for vitamin D deficiency and consequently dyslipidemia. Second, those with lower BMI, particularly underweight individuals, may experience vitamin D deficiency and other nutritional deficiencies due to imbalanced diets or inadequate nutrient intake. This state of nutrient deficiency could further impact lipid metabolism, elevating the risk of dyslipidemia. Finally, individuals with a family history of hyperlipidemia may inherit gene variants that affect vitamin D or lipid metabolism, rendering them more vulnerable to both vitamin D deficiency and dyslipidemia (62). We intend to explore these potential factors further to develop a more comprehensive predictive model for hyperlipidemia risk.

In this study, a threshold of 30 ng/mL was set for vitamin D levels, and significant differences in the prevalence of dyslipidemia and individual lipid profile measures were observed between the two groups. Even after adjusting for various potential confounders, vitamin D deficiency remained significantly associated with an elevated risk of dyslipidemic events. This finding aligns with prior meta-analyses (63) and individual studies (64–66), which collectively indicate a close relationship between serum vitamin D levels and lipid markers such as TC, TG, LDL-C, and HDL-C. An increase in serum 25(OH)D levels has been shown to aid in reducing blood lipid levels and improving dyslipidemia (67). Although adjustments were made for multiple traditional risk factors, residual confounders such as dietary habits, genetic background, and medication use may still influence the relationship between vitamin D levels and hyperlipidemia (68, 69). Insufficient intake of vitamin D-rich foods, such as fish, dairy products, and fortified foods, may contribute to vitamin D deficiency. Concurrently, diets high in fat, sugar, and salt could exacerbate lipid metabolism disorders, increasing the risk of dyslipidemia. Additionally, genetic background plays a key role in both vitamin D metabolism and lipid metabolism. Genetic variants may influence the activity of vitamin D receptors or related enzymes, affecting vitamin D bioavailability and lipid processing. Furthermore, medication use is a critical factor impacting both vitamin D levels and lipid abnormalities; certain drugs, such as antiepileptics, antibiotics, and lipid-lowering agents, may alter vitamin D metabolism or elevate the risk of dyslipidemia. Given individual variability and the complexity of these interactions, unidentified or insufficiently understood confounders may still impact the association between vitamin D levels and hyperlipidemia. Future studies should more deeply explore these potential factors and utilize more precise study designs and sensitive detection methods to uncover the subtler relationships between vitamin D and lipid abnormalities.

Given the extensive physiological effects of vitamin D (70), particularly its pivotal role in metabolic regulation, we emphasize the importance of ensuring adequate vitamin D levels. The long half-life of vitamin D, which allows it to persist in the human body for 3 weeks and maintain a relatively stable blood concentration, provides a rationale for its use as a mild and effective control measure (56). Consequently, it is reasonable to encourage patients to ensure sufficient vitamin D intake during glycemic and lipid-lowering therapy (27). However, there is currently a lack of support from large-scale, long-term studies regarding the optimal dosage and frequency of vitamin D supplementation, necessitating cautious consideration of individual differences and potential risks when formulating supplementation strategies (71). Our study has made certain progress in exploring the relationship between serum 25(OH)D levels and the risk of hyperlipidemia in adults. Its strengths lie in the rigorous design, employing a cohort study approach, which enables the observation of the impact of 25(OH)D levels over time on hyperlipidemia risk. Furthermore, the study considered various potential confounding factors, such as age, gender, and BMI, thereby enhancing the accuracy and reliability of the findings. Nonetheless, our study also has some limitations. Firstly, during data collection, factors that may influence vitamin D levels, such as habitual diet, sun exposure time, and sunscreen use, were not considered. These factors could potentially interfere with the study results and affect the accuracy of the conclusions. Secondly, the study sample was regionally limited, and the follow-up period was relatively short, which may impact the reliability of the correlation test between 25(OH)D and hyperlipidemia. This limitation may be particularly evident in subgroup analyses, challenging the robustness of the conclusions. Additionally, the insufficient sample size and follow-up duration may restrict the generality and applicability of the study results. Future research is needed to conduct a series of targeted studies to clarify the recommended dosage and frequency of vitamin D supplements, maximizing their benefits while minimizing potential risks. It is essential to identify target populations, such as elderly individuals and specific high-risk groups (e.g., obese and diabetic patients), who are likely to benefit most from these interventions, in order to provide more precise vitamin D management guidelines to support personalized treatment in clinical practice. At the same time, we also need to consider factors such as habitual diet, sunshine time, and sunscreen use into the study to more comprehensively and accurately assess the effect of vitamin D on the risk of hyperlipidemia. This study also provides some implications for enhancing the intake of vitamin D-rich foods such as milk, salted fish and cod liver oil (72). By continuously exploring and optimizing vitamin D management strategies, we are expected to make a greater contribution to improving metabolic health and preventing chronic diseases. Future studies need to further increase the sample size and prolong the follow-up time, so as to improve the accuracy and reliability of the study and provide more strong evidence support for clinical practice.

## 5 Conclusion

This study is a three-year prospective cohort analysis conducted in Zhejiang, which delves into the relationship between serum 25(OH)D levels and the risk of hyperlipidemia in adults. The results indicate a significant correlation between vitamin D status and the incidence of hyperlipidemia, and this association remains robust even after extensive adjustment for confounding factors, further confirming the crucial role of vitamin D in lipid metabolism. Women are more prone to vitamin D deficiency compared to men, and this gender disparity may be associated with factors such as lifestyle, physiological characteristics, and occupational activities. Additionally, older age, lower educational attainment, lower BMI, and a family history are predictive of both vitamin D deficiency and dyslipidemia. Compared to existing research, this study not only validates the close correlation between vitamin D and lipid levels but also uncovers the distribution characteristics of vitamin D deficiency across different populations and its independent impact on the risk of hyperlipidemia. Future research should further explore the optimal dosage and frequency of vitamin D supplementation and how to optimize vitamin D management strategies for specific high-risk populations to improve metabolic health, providing novel insights into the prevention and treatment of hyperlipidemia. The findings of this study offer compelling evidence for the potential role of vitamin D in the prevention and control of hyperlipidemia, emphasizing the importance of maintaining adequate vitamin D levels.

## Data Availability

The datasets presented in this study can be found in online repositories. The names of the repository/repositories and accession number(s) can be found in the article/supplementary material.

## References

[1] Li N. Platelets as an inter-player between hyperlipidaemia and atherosclerosis. J Intern Med. (2024) 296:39–52. doi: 10.1111/joim.13794, PMID: 38704820

[2] Nohria A Shah JT Desai D Alhanshali L Ingrassia J Femia A. Alopecia areata and cardiovascular comorbidities: a cross-sectional analysis of the all of us research program. JAAD Int. (2024) 16:46–8. doi: 10.1016/j.jdin.2024.03.024, PMID: 38774345 PMC11107229

[3] Faluk M Wardhere A Mohamoud A Nor M Bampastsias D Oikonomou E. Evolution of LDL-C lowering medications and their cardiovascular benefits: past, present, and future. Curr Probl Cardiol. (2024) 49:102637–7. doi: 10.1016/j.cpcardiol.2024.102637, PMID: 38735347

[4] Yang M Song Z Wang S He Y Wang W Gong H. Protective effect of bamboo root dietary fibre on hyperlipidaemia mice induced by high-fat diet. CYTA J Food. (2023) 21:674–81. doi: 10.1080/19476337.2023.2274371

[5] Takaoka M Zhao X Lim HY Magnussen CG Ang O Suffee N. Early intermittent hyperlipidaemia alters tissue macrophages to fuel atherosclerosis. Nature. (2024) 634:457–65. doi: 10.1038/s41586-024-07993-x, PMID: 39231480 PMC11464399

[6] Huang X Hui H Zhu W Chen N Wei Y Wang Z. Effect of the interaction between alcohol and meat consumption on the hyperlipidaemia risk among elderly individuals: evidence from Shanghai, China. Front Nutr. (2022) 9:982626–6. doi: 10.3389/fnut.2022.982626, PMID: 36324622 PMC9618893

[7] Vardell E. Global Health Observatory data repository. Med Ref Serv Q. (2020) 39:67–74. doi: 10.1080/02763869.2019.1693231 32069199

[8] McDermott CM Feola R Plude J. Detection of cyanobacterial toxins (microcystins) in waters of northeastern Wisconsin by a new immunoassay technique. Toxicon. (1995) 33:1433–42. doi: 10.1016/0041-0101(95)00095-4, PMID: 8744983

[9] Mohamed-Yassin M-S Rosman N Kamaruddin KN Miptah HN Baharudin N Ramli AS. A systematic review and meta-analysis of the prevalence of dyslipidaemia among adults in Malaysia. Sci Rep. (2023) 13:11036–6. doi: 10.1038/s41598-023-38275-7, PMID: 37419924 PMC10328969

[10] Meza-Meza MR Ruiz-Ballesteros AI de la Cruz-Mosso U. Functional effects of vitamin D: from nutrient to immunomodulator. Crit Rev Food Sci Nutr. (2022) 62:3042–62. doi: 10.1080/10408398.2020.1862753 33354999

[11] Carlberg C. Nutrigenomics of vitamin D. Nutrients. (2019) 11:676. doi: 10.3390/nu11030676, PMID: 30901909 PMC6470874

[12] Bouillon R Carmeliet G Verlinden L van Etten E Verstuyf A Luderer HF. Vitamin D and human health: lessons from vitamin D receptor null mice. Endocr Rev. (2008) 29:726–76. doi: 10.1210/er.2008-0004, PMID: 18694980 PMC2583388

[13] Van Etten E Mathieu C. Immunoregulation by 1,25-dihydroxyvitamin D3: basic concepts. J Steroid Biochem Mol Biol. (2005) 97:93–101. doi: 10.1016/j.jsbmb.2005.06.002 16046118

[14] Martinelli RP Rayego-Mateos S Alique M Márquez-Expósito L Tejedor-Santamaria L Ortiz A. Vitamin D, cellular senescence and chronic kidney diseases: what is missing in the equation? Nutrients. (2023) 15:1349. doi: 10.3390/nu15061349, PMID: 36986078 PMC10056834

[15] Kim D-H Meza CA Clarke H Kim J-S Hickner RC. Vitamin D and endothelial function. Nutrients. (2020) 12:575. doi: 10.3390/nu12020575, PMID: 32098418 PMC7071424

[16] Borges MC Martini LA Rogero MM. Current perspectives on vitamin D, immune system, and chronic diseases. Nutrition. (2011) 27:399–404. doi: 10.1016/j.nut.2010.07.022, PMID: 20971616

[17] Soto-Dávila M Valderrama K Inkpen SM Hall JR Rise ML Santander J. Effects of vitamin D 2 (Ergocalciferol) and D 3 (cholecalciferol) on Atlantic Salmon (*Salmo salar*) primary macrophage immune response to *Aeromonas salmonicida* subsp *salmonicida* infection. Front Immunol. (2020) 10:3011. doi: 10.3389/fimmu.2019.03011, PMID: 32010129 PMC6973134

[18] Rajakumar K de las Heras J Chen TC Lee SJ Holick MF Arslanian SA. Vitamin D status, adiposity, and lipids in black American and Caucasian children. J Clin Endocrinol Metab. (2011) 96:1560–7. doi: 10.1210/jc.2010-2388, PMID: 21367931 PMC3085205

[19] Huang X Yang Y Jiang Y Zhou Z Zhang J. Association between vitamin D deficiency and lipid profiles in overweight and obese adults: a systematic review and meta-analysis. BMC Public Health. (2023) 23:1653–3. doi: 10.1186/s12889-023-16447-4, PMID: 37644450 PMC10464009

[20] Alzohily B AlMenhali A Gariballa S Munawar N Yasin J Shah I. Unraveling the complex interplay between obesity and vitamin D metabolism. Sci Rep. (2024) 14:7583–3. doi: 10.1038/s41598-024-58154-z, PMID: 38555277 PMC10981658

[21] Song X Qin S Chen S Zhang C Lin L Song Z. Bibliometric analysis of vitamin D and obesity research over the period 2000 to 2023. Front Pharmacol. (2024) 15:1445061. doi: 10.3389/fphar.2024.1445061, PMID: 39092232 PMC11291317

[22] Di Filippo L De Lorenzo R Giustina A Rovere-Querini P Conte C. Vitamin D in osteosarcopenic obesity. Nutrients. (2022) 14:1816. doi: 10.3390/nu14091816, PMID: 35565781 PMC9100750

[23] Song S Yuan Y Wu X Zhang D Qi Q Wang H. Additive effects of obesity and vitamin D insufficiency on all-cause and cause-specific mortality. Front Nutr. (2022) 9:999489–9. doi: 10.3389/fnut.2022.999489, PMID: 36337642 PMC9634746

[24] Ponda MP Dowd K Finkielstein D Holt PR Breslow JL. The short-term effects of vitamin D repletion on cholesterol: a randomized, placebo-controlled trial. Arterioscler Thromb Vasc Biol. (2012) 32:2510–5. doi: 10.1161/ATVBAHA.112.254110, PMID: 22947589 PMC3633472

[25] Lu L Yu Z Lin X. Plasma 25-Hydroxyvitamin D concentration and metabolic syndrome among middle-aged and elderly Chinese individuals. Diabetes Care. (2010) 33:e14. doi: 10.2337/dc09-1713 19366976 PMC2699709

[26] Miñambres I Sánchez-Quesada JL Sánchez-Hernández J Rodríguez J de Leiva A Pérez A. Vitamin D concentrations in familial combined hyperlipidemia: effects of lipid lowering treatment. Diabetol Metab Syndr. (2014) 6:7. doi: 10.1186/1758-5996-6-7, PMID: 24450309 PMC3904931

[27] Al-Qusous MN Al Madanat WKJ Mohamed Hussein R. Association of Vitamins D, B6, and B12 deficiencies with hyperlipidemia among Jordanian adults. Rep Biochem Mol Biol. (2023) 12:415–24. doi: 10.61186/rbmb.12.3.415 38618263 PMC11015927

[28] Yang Y Yan S Yao N Guo Y Wang H Sun M. Effects of vitamin D supplementation on the regulation of blood lipid levels in prediabetic subjects: a meta-analysis. Front Nutr. (2023) 10:983515–5. doi: 10.3389/fnut.2023.983515, PMID: 36969817 PMC10033891

[29] Harreiter J Mendoza LC Simmons D Desoye G Devlieger R Galjaard S. Vitamin D3 supplementation in overweight/obese pregnant women: no effects on the maternal or fetal lipid profile and body fat distribution-a secondary analysis of the multicentric, randomized, controlled vitamin D and lifestyle for gestational diabetes prevention trial (DALI). Nutrients. (2022) 14:3781. doi: 10.3390/nu14183781, PMID: 36145157 PMC9503968

[30] Li J-M Yang H-Y Wu S-H Dharmage SC Jalaludin B Knibbs LD. The associations of particulate matter short-term exposure and serum lipids are modified by vitamin D status: a panel study of young healthy adults. Environ Pollut. (2023) 317:120686. doi: 10.1016/j.envpol.2022.120686 36400145

[31] Alsufiani HM AlGhamdi SA AlShaibi HF Khoja SO Saif SF Carlberg C. A single vitamin D3 bolus supplementation improves vitamin D status and reduces proinflammatory cytokines in healthy females. Nutrients. (2022) 14:3963. doi: 10.3390/nu14193963, PMID: 36235615 PMC9570631

[32] Zhu JG. Guidelines for prevention and treatment of dyslipidemia in Chinese adults (2016 revised edition). Chin Circulat J. (2016) 31:937–53.

[33] Phanmaneelux N Buranakitjaroen P Kunanon S Roubsanthisuk W Chotruangnapa C. The ASSOCIATION between hypertension subtypes defined by home blood pressure in hypertensive patients and incidence of hypertension mediated organ damage. J Hypertens. (2024) 42:e203. doi: 10.1097/01.hjh.0001021500.24571.d8

[34] de Waal E Pienaar AE. Influences of persistent overweight on perceptual-motor proficiency of primary school children: the north-west CHILD longitudinal study. BMC Pediatr. (2021) 21:245–10. doi: 10.1186/s12887-021-02708-x, PMID: 34016074 PMC8136082

[35] Stach K Richter H Fraass U Stein A. Quantifying the “distance to LDC-goal” in patients at very high cardiovascular risk with hyperlipidaemia in Germany: a retrospective claims database analysis. Ther Adv Cardiovasc Dis. (2024) 18:17539447241277402. doi: 10.1177/17539447241277402, PMID: 39340274 PMC11440620

[36] Chen Zengli JY Yutong L Jie L Shiqin L Mingting L. Dyslipidemia prevalence in Chinese older adults: a Meta-analysis. Zhongguo Quanke Yixue. (2022) 25:115–21. doi: 10.12114/j.issn.1007-9572.2021.00.328

[37] van den Heuvel EG Lips P Schoonmade LJ Lanham-New SA van Schoor NM. Comparison of the effect of daily vitamin D2 and vitamin D3 supplementation on serum 25-Hydroxyvitamin D concentration (Total 25(OH)D, 25(OH)D2, and 25(OH)D3) and importance of body mass index: a systematic review and Meta-analysis. Adv Nutr. (2024) 15:100133. doi: 10.1016/j.advnut.2023.09.016, PMID: 37865222 PMC10831883

[38] Liu C Chen L. Letter to the editor, “effects of vitamin D deficiency on blood lipids and bone metabolism: a large cross-sectional study.”. J Orthop Surg Res. (2023) 18:307–7. doi: 10.1186/s13018-023-03773-x, PMID: 37072808 PMC10111671

[39] Herrmann M Keppel M Zelzer S Pilz S Scharnagl H Kleber M. The role of functional vitamin D deficiency and low vitamin D reservoirs in relation to cardiovascular health and mortality. Clin Chim Acta. (2024) 558:118230. doi: 10.1016/j.cca.2024.118230 38890759

[40] Ren C Zhang S Hong B Guan L Huang W Feng J. Germinated brown rice relieves hyperlipidemia by alleviating gut microbiota dysbiosis. J Integr Agric. (2023) 22:945–57. doi: 10.1016/j.jia.2023.02.015

[41] Xie Z Li E Gao G Du Y Wang M Wang H. Zexie tang targeting FKBP38/mTOR/SREBPs pathway improves hyperlipidemia. J Ethnopharmacol. (2022) 290:115101–1. doi: 10.1016/j.jep.2022.115101, PMID: 35151834

[42] Karr S. Epidemiology and management of hyperlipidemia. Am J Manag Care. (2017) 23:S139–48. 28978219

[43] Wan Z Guo J Pan A Chen C Liu L Liu G. Association of Serum 25-Hydroxyvitamin D concentrations with all-cause and cause-specific mortality among individuals with diabetes. Diabetes Care. (2021) 44:350–7. doi: 10.2337/dc20-1485, PMID: 33168652

[44] Li X. The FGF metabolic axis. Front Med. (2019) 13:511–30. doi: 10.1007/s11684-019-0711-y, PMID: 31495905 PMC7102389

[45] Harahap IA Landrier J-F Suliburska J. Interrelationship between vitamin D and calcium in obesity and its comorbid conditions. Nutrients. (2022) 14:3187. doi: 10.3390/nu14153187, PMID: 35956362 PMC9370653

[46] Ruiz-Ojeda FJ Anguita-Ruiz A Leis R Aguilera CM. Genetic factors and molecular mechanisms of vitamin D and obesity relationship. Ann Nutr Metab. (2018) 73:89–99. doi: 10.1159/000490669, PMID: 29982250

[47] Riek AE Oh J Bernal-Mizrachi C. Vitamin D regulates macrophage cholesterol metabolism in diabetes. J Steroid Biochem Mol Biol. (2010) 121:430–3. doi: 10.1016/j.jsbmb.2010.03.018, PMID: 20338238

[48] Li C Fu J Ye Y Li J He Y Fang T. The impact of vitamin D on the etiopathogenesis and the progression of type 1 and type 2 diabetes in children and adults. Front Endocrinol. (2024) 15:1360525–5. doi: 10.3389/fendo.2024.1360525, PMID: 38650715 PMC11033370

[49] Pittas AG Jorde R Kawahara T Dawson-Hughes B. Vitamin D supplementation for prevention of type 2 diabetes mellitus: to D or not to D? J Clin Endocrinol Metab. (2020) 105:3721–33. doi: 10.1210/clinem/dgaa594, PMID: 32844212 PMC7571449

[50] Gagnon C Lu ZX Magliano DJ Dunstan DW Shaw JE Zimmet PZ. Low serum 25-Hydroxyvitamin D is associated with increased risk of the development of the metabolic syndrome at five years: results from a national, population-based prospective study (The Australian diabetes, obesity and lifestyle study: AusDiab). J Clin Endocrinol Metab. (2012) 97:1953–61. doi: 10.1210/jc.2011-3187, PMID: 22442263

[51] Muscella A Stefàno E Lunetti P Capobianco L Marsigliante S. The regulation of fat metabolism during aerobic exercise. Biomolecules. (2020) 10:1699. doi: 10.3390/biom10121699, PMID: 33371437 PMC7767423

[52] Janssen HCJP Emmelot-Vonk MH Verhaar HJJ van der Schouw YT. Determinants of vitamin D status in healthy men and women aged 40–80 years. Maturitas. (2013) 74:79–83. doi: 10.1016/j.maturitas.2012.10.008, PMID: 23200514

[53] Zaydieva YZ Balan VE. Vitamin D and women’s reproductive health (literature review). Medicinskij Sovet. (2018):164–72. doi: 10.21518/2079-701X-2018-12-164-172

[54] Ciarambino T Crispino P Minervini G Giordano M. Vitamin D: can gender medicine have a role? Biomedicines. (2023) 11:1762. doi: 10.3390/biomedicines11061762, PMID: 37371857 PMC10296422

[55] Lupton JR Faridi KF Martin SS Sharma S Kulkarni K Jones SR. Deficient serum 25-hydroxyvitamin D is associated with an atherogenic lipid profile: The very large database of lipids (VLDL-3) study. J Clin Lipidol. (2016) 10:72–81.e1. doi: 10.1016/j.jacl.2015.09.006, PMID: 26892123 PMC4762185

[56] Jorde R Figenschau Y Hutchinson M Emaus N Grimnes G. High serum 25-hydroxyvitamin D concentrations are associated with a favorable serum lipid profile. Eur J Clin Nutr. (2010) 64:1457–64. doi: 10.1038/ejcn.2010.176, PMID: 20823896

[57] Giustina A Bouillon R Dawson-Hughes B Ebeling PR Lazaretti-Castro M Lips P. Vitamin D in the older population: a consensus statement. Endocrine. (2023) 79:31–44. doi: 10.1007/s12020-022-03208-3, PMID: 36287374 PMC9607753

[58] Qorbani M Zarei M Moradi Y Appannah G Djalainia S Pourrostami K. Effect of vitamin D supplementation on cardiac-metabolic risk factors in elderly: a systematic review and meta-analysis of clinical trials. Diabetol Metab Syndr. (2022) 14:88–8. doi: 10.1186/s13098-022-00859-0, PMID: 35752843 PMC9233853

[59] Tanaka K Ao M Tamaru J Kuwabara A. Vitamin D insufficiency and disease risk in the elderly. J Clin Biochem Nutr. (2024) 74:9–16. doi: 10.3164/jcbn.23-59, PMID: 38292127 PMC10822750

[60] Wyskida M Wieczorowska-Tobis K Chudek J. Prevalence and factors promoting the occurrence of vitamin D deficiency in the elderly. Postepy Hig Med Dosw. (2017) 71:01–204. doi: 10.5604/01.3001.0010.3804 28345527

[61] Harvey NC Ward KA Agnusdei D Binkley N Biver E Campusano C. Optimisation of vitamin D status in global populations. Osteoporos Int. (2024) 35:1313–22. doi: 10.1007/s00198-024-07127-z, PMID: 38836946

[62] Mendes MM Gomes APO Araújo MM Coelho ASG Carvalho KMB Botelho PB. Prevalence of vitamin D deficiency in South America: a systematic review and meta-analysis. Nutr Rev. (2023) 81:1290–309. doi: 10.1093/nutrit/nuad010, PMID: 36882047

[63] Dibaba DT. Effect of vitamin D supplementation on serum lipid profiles: a systematic review and meta-analysis. Nutr Rev. (2019) 77:890–902. doi: 10.1093/nutrit/nuz037 31407792

[64] Chen X Zhou M Yan H Chen J Wang Y Mo X. Association of serum total 25-hydroxy-vitamin D concentration and risk of all-cause, cardiovascular and malignancies-specific mortality in patients with hyperlipidemia in the United States. Front Nutr. (2022) 9:971720–13. doi: 10.3389/fnut.2022.971720, PMID: 36337630 PMC9631937

[65] Liu W Wu Z Zhu D Chen G Yan G Zhang S. Vitamin D and lipid profiles in postmenopausal women: a meta-analysis and systematic review of randomized controlled trials. Front Mol Biosci. (2021) 8:799934–4. doi: 10.3389/fmolb.2021.799934, PMID: 34977158 PMC8719197

[66] Williams DM Fraser A Lawlor DA. Associations of vitamin D, parathyroid hormone and calcium with cardiovascular risk factors in US adolescents. Heart. (2011) 97:315–20. doi: 10.1136/hrt.2010.203224, PMID: 21193684 PMC3931194

[67] Zittermann A Trummer C Theiler-Schwetz V Lerchbaum E März W Pilz S. Vitamin D and cardiovascular disease: an updated narrative review. Int J Mol Sci. (2021) 22:2896. doi: 10.3390/ijms22062896, PMID: 33809311 PMC7998446

[68] Lee SS Ling KH Tusimin M Subramaniam R Rahim KF Loh SP. Interplay between maternal and neonatal vitamin D deficiency and vitamin-D-related gene polymorphism with neonatal birth anthropometry. Nutrients. (2022) 14:564. doi: 10.3390/nu14030564, PMID: 35276923 PMC8839863

[69] Lin S Li J Zhang Y Song X Chen G Pei L. Maternal passive smoking, vitamin D deficiency and risk of spontaneous abortion. Nutrients. (2022) 14:3674. doi: 10.3390/nu14183674, PMID: 36145050 PMC9501103

[70] Khoshnaw M Dizaye K. Beneficial effects of vitamin D in the management of untreated hyperlipidemia in diabetic patients in Erbil, Iraq. Farmacija. (2022) 69:847–53. doi: 10.3897/pharmacia.69.e90908

[71] Jaun F Boesing M Luethi-Corridori G Abig K Bloch N Giezendanner S. Effect of single high dose vitamin D substitution in hospitalized COVID-19 patients with vitamin D deficiency on length of hospital stay. Biomedicines. (2023) 11:1277. doi: 10.3390/biomedicines11051277, PMID: 37238948 PMC10215464

[72] Pludowski P. Supplementing vitamin D in different patient groups to reduce deficiency. Nutrients. (2023) 15:3725. doi: 10.3390/nu15173725, PMID: 37686757 PMC10489803

[73] Patriota P Rezzi S Guessous I Marques-Vidal P. Vitamin D and Weight Change: A Mendelian Randomization, Prospective Study. International journal of Molecular Sciences. (2022) 23:11100. doi: 10.3390/ijms231911100, PMID: 36232402 PMC9569579

[74] Rajakumar K Moore CG Khalid AT Vallejo AN Virji MA Holick MF. Effect of vitamin D3 supplementation on vascular and metabolic health of vitamin D–deficient overweight and obese children: a randomized clinical trial. The American Journal of Clinical Nutrition. (2020) 111:757–768. doi: 10.1093/ajcn/nqz340, PMID: 31950134 PMC7138671

[75] Mead MJ McWhorter CA Rodgers MD Ebeling MD Shary JR Gregoski MJ. Does maternal vitamin D status influence placental weight or vascular and inflammatory pathology? Secondary analysis from the Kellogg Pregnancy Study. The Journal of steroid biochemistry and molecular biology. (2023) 233:106358–106358. doi: 10.1016/j.jsbmb.2023.106358, PMID: 37414103 PMC11229515

